# Regulation of SIRT1 in Vascular Smooth Muscle Cells from Streptozotocin-Diabetic Rats

**DOI:** 10.1371/journal.pone.0065666

**Published:** 2013-05-29

**Authors:** Alice Toniolo, Erica Alessia Warden, Alberto Nassi, Andrea Cignarella, Chiara Bolego

**Affiliations:** Department of Pharmaceutical and Pharmacological Sciences, University of Padova, Padova, Italy; Pennington Biomedical Research Center, United States of America

## Abstract

Sirtuins enzymes are a conserved family of nicotinamide adenine dinucleotide (NAD)-dependent deacetylases and ADP-ribosyltransferases that mediate responses to oxidative stress, fasting and dietary restriction in mammals. Vascular smooth muscle cells (VSMCs) are involved in many mechanisms that regulate vascular biology *in vivo* but the role of SIRT1 has not been explored in much detail. Therefore, we investigated the regulation of SIRT1 in cultured VSMCs under various stress conditions including diabetes. Sprague-Dawley rats were made diabetic by injecting a single dose of streptozotocin (65 mg/Kg), and aortic VSMCs were isolated after 4 weeks. Immunocytochemistry showed that SIRT1 was localized predominantly in the nucleus, with lower staining in VSMCs from STZ-diabetic as compared with normoglycemic rats. Previous diabetes induction *in vivo* and high glucose concentrations *in vitro* significantly downregulated SIRT1 amounts as detected in Western blot assays, whereas TNF-α (30 ng/ml) stimulation failed to induce significant changes. Because estrogen signaling affects several pathways of oxidative stress control, we also investigated SIRT1 modulation by 17β-estradiol. Treatment with the hormone (10 nM) or a selective estrogen receptor-α agonist decreased SIRT1 levels in VSMCs from normoglycemic but not in those from STZ-diabetic animals. 17β-estradiol treatment also enhanced activation of AMP-dependent kinase, which partners with SIRT1 in a signaling axis. SIRT1 downregulation by 17β-estradiol could be observed as well in human peripheral blood mononuclear cells, a cell type in which SIRT1 downregulation is associated with insulin resistance and subclinical atherosclerosis. These data suggest that SIRT1 protein levels are regulated by diverse cellular stressors to a variable extent in VSMCs from diabetic and normoglycemic rats, warranting further investigation on SIRT1 as a modulator of VSMC activity in settings of vascular inflammation.

## Introduction

Vascular aging is characterized by increased oxidative stress and proinflammatory phenotypic alterations. Metabolic stress, such as chronic hyperglycemia in diabetes, is known to increase the production of reacting oxygen species (ROS) and promote inflammatory gene expression, accelerating vascular aging [1]. Vascular smooth muscle cells (VSMCs) are sensitive to inflammatory lesions, and notable responses thereof such as proliferation and migration are accompanied by enhanced expression of proinflammatory cytokines, especially TNF-α. Agents endowed with inhibitory effects on VSMC responses such as those underlying neointima formation may be suitable for intervention in vascular disease [2].

Silent information regulator of gene transcription (SIRT)1 is a prominent member of a family of NAD-dependent enzymes and affects a variety of cellular functions ranging from gene silencing, regulation of cell cycle and apoptosis to energy homeostasis. Use of cell models as well as tissue-specific SIRT1 knockout mice has uncovered potential roles for SIRT1 in disease settings such as diabetes and cardiovascular disease, inflammation, neurodegeneration and cancer [3]. Several recent studies have implicated SIRT1 in the regulation of inflammatory responses. Whereas caloric restriction enhances SIRT1 activity, hyperglycemia induces vascular cell senescence by reducing SIRT1 activity and thereby contribute to the development of diabetic vascular dysfunction. Hyperglycemia decreases SIRT1 expression in cultured endothelial cells [4], whereas overexpression of SIRT1 prevents the hyperglycemia-induced vascular cell senescence and thereby protects against vascular dysfunction in mice with diabetes [4], [5]. SIRT1 is expressed not only in the endothelium but also in VSMCs [2], [6], [7], where it is required for both growth and proliferation, suggesting a potential role of SIRT1 in the control of vascular function under various stress stimuli.

While the preponderance of genetic data indicates that increasing SIRT1 levels or its activity has beneficial physiological effects [8], reports are sometimes conflicting [9]. For instance, the pharmacological inhibition of sirtuin decreased the production of inflammatory cytokines in LPS-stimulated macrophages [10]. In addition, limited information is available as to SIRT1 produced in vascular smooth muscle cells and the modulation thereof by inflammatory and/or metabolic factors. Thus, as SIRT1 appears to be strategically involved in many mechanisms that regulate vascular biology *in vivo*, objective of this work was to investigate its regulation in cultured VSMCs under various stress conditions. Because a protective role of SIRT1 has been suggested in the pathogenesis of diabetic vasculopathy [4], [5], we determined SIRT1 levels in VSMCs from normoglycemic and diabetic rats, as well as under inflammatory conditions such as after incubation with the pro-inflammatory cytokine TNF-α. Because of the functional correlation between SIRT1 and estrogen signaling in cancer cells [11], we also assessed SIRT1 modulation by 17β-estradiol in rat VSMCs and, for comparison, in human peripheral blood mononuclear cells (PBMCs), a cell type in which SIRT1 downregulation is associated with insulin resistance and subclinical atherosclerosis [12].

## Materials and Methods

### Ethics statement

The principles of laboratory care relating to the protection and welfare of vertebrates used for scientific purposes issued by the Directive 2010/63/EU were followed in this study, which was approved by the Institutional Animal Care and Use Committee (CEASA based on the Italian acronym) at the University of Padova. Rats were anesthetized by i.m. injection of Zoletil (tiletamine HCl and zolazepam HCl) before diabetes induction, and sacrificed under euthanizing pentobarbital (150 mg/kg i.p.) anesthesia.

### Diabetes induction

Adult Sprague-Dawley rats weighing 200±14 g (2-months-old) were used for inducing diabetes. The animals were injected i.v. with streptozotocin (STZ) at the dose of 65 mg/kg body weight; control animals were injected with the vehicle and kept in a separate cage. During the 24 hours after the induction, to avoid hypoglycemic crisis, drinking water was substituted with 5% glucose solution. Four days later glycosuria was tested to confirm diabetes induction. At sacrifice 4 weeks after STZ injection, animals had plasma glucose levels above 25 mM (300 mg/dl). STZ-treated animals show various characteristics observed in untreated diabetic subjects such as hyperglycemia, polydipsia, polyuria and loss of weight [13].

### Cell cultures

VSMCs were obtained from aortic intimal-medial layers of Sprague-Dawley rats and cultured as described previously [14], [15]. In brief, VSMCs at passage 5 were switched to phenol red-free M199 with 10% charcoal-stripped fetal calf serum (FCS) for 48 h. Thereafter, they were synchronized in medium containing 0.4% FCS for 24 h and incubated as described.

Human lympho-monocytes were isolated from whole blood or buffy coat provided by the Transfusion Unit at Padua University Hospital. Blood was diluted 1∶3 with M 199 medium in a 50-ml Falcon tube and separated from cells using Ficoll 1077 (15 ml blood: 20 ml Ficoll); this solution was centrifuged at 100 *g* for 30 min. The lympho-monocyte ring was isolated and transferred into a new Falcon tube, suspended in 50 ml of fresh M199 and centrifuged again for 20 min. Supernatants were removed, and for other two times cells were washed with 50 ml of M199 and centrifuged at 50 *g* for 15 min. The final pellet was resuspended in RPMI 1640 culture medium supplemented with 20% FBS, 2 mmol/l glutamine and 0.5% penicillin-streptomycin, and cells seeded in 6-well plates (2 ml/well). Monocytes adhere to the well bottom after 2 h from seeding, thus the culture medium was refreshed and non-adhered cells were removed.

### Cell treatment

Plated VSMCs (3×10^5^/well) were incubated with 10% FBS culture medium until subconfluence, washed three times with PBS and incubated with 0.4% FBS medium for 24 h to arrest cell growth and synchronize them all to the same phase of the cell cycle. After synchronization, VSMCs were washed once with PBS to remove any trace of FBS and stimulated for different lengths of time with culture medium supplemented with 2% FBS. Based on the experimental design, cells were stimulated with TNF-α (30 ng/ml); D-(+)-glucose (3 g/l) to mimic *in vitro* the diabetic condition of hyperglycemia; or estrogen (10 nM), which requires a specific phenol red-free culture medium because phenol red has weak estrogenic activity and could interfere with the correct course of the stimulation. The selective estrogen receptor-α agonist PPT and the selective estrogen receptor-β agonist DPN were purchased from Tocris Biosciences (Bristol, UK). Control VSMCs were incubated with 2% FBS culture medium only. At the end of incubations, cells were washed twice with cold PBS, scraped off and harvested in 100 µl of lysis buffer [16] consisting of 50 mM Tris HCl pH 7.4, 150 mM NaCl, 1% NP-40, 25 mM NaF, 0.5% sodium deoxycholate, 10% SDS, 1 mM EGTA, 1 mM sodium orthovanadate, 10 mM sodium pyrophosphate, 1 mM phenyl-methyl-sulfonyl-fluoride and Complete protease inhibitors (Roche). To achieve complete lysis, cell samples underwent four freezing/thaw cycles and were stored at –20°C until analysis.

### Immunocytochemistry

VSMCs were seeded at the 6^th^ passage in 24-well plates (roughly 30,000/well) on a sterile cover slip previously placed on the well bottom. Two days after plating, culture medium was removed and cells were washed twice with sterile PBS to remove any residue of the medium. Cells were then fixed with 4% paraformaldehyde for 10 min at room temperature, and washed three times with sterile PBS to remove the excess of paraformaldehyde. In order to saturate aspecific binding sites, cells were blocked for 45 min with a permeabilization solution of PBS supplemented with a small amount of FBS and 0.1% Triton. Subsequently, the solution was removed and without washing, the primary antibody was added in each well: mouse anti-α-actin, typical protein expressed by VSMCs, which is linked to phycoerythrin, a fluorescent probe (150 µl/well, 1∶400 in PBS containing 2% FBS) and cells were incubated for 1 h at room temperature. At the end of the incubation, cells were washed three times with a PBS solution plus RNAse (1∶40) for 15 min at 37°C, so as to lyse RNA in the nuclei. Cells were then washed with distilled water to remove excess RNAse and ease the removal of cover slips. The drop of Mowiol mounting media on the slip holder was added of propidium iodide, previously diluted in distilled water (1∶10, final concentration: 0.5 mg/ml): this agent is able to penetrate the nuclei and label them in red. The cover slips were removed and turned over Mowiol and at last stored at 4°C away from light until image acquisition using a confocal microscope. Cell fluorescence was measured using ImageJ.

### Western blotting

Whole cell lysates (30 µg) were resolved by 10% SDS-PAGE. The same protein amount was loaded for each sample. Proteins were transferred onto a polyvinilidene difluoride (PVDF) membrane in a cold buffer containing 25 mM Tris, 192 nM glycine and 20% methanol. After blocking with Tris buffered saline (TBS: 1 M Tris HCl pH 7.4, 5 M NaCl, 0.1% Tween 20) added of 5% non-fat milk (to saturate aspecific binding sites) for 1 h, membranes were incubated with the primary antibodies for SIRT1 (1∶500 dilution, Abcam), p-AMPK and AMPK (both from Abcam) overnight at 4° C. The following day, the membranes were washed of excess primary antibody with TBS and incubated for 45 min with horseradish peroxidase-conjugated anti-rabbit immunoglobulin antibody. Specific proteins were detected using enhanced chemiluminescence (ECL1 and ECL2, GE Healthcare). Band intensities were quantified by densitometry and the results were normalized to β-actin (specific antibody diluted 1∶5000, Sigma-Aldrich).

### Statistical analysis

Results were expressed as the mean ± SEM of multiple independent experiments for in vitro assays. The Student *t* test was used to compare 2 groups or ANOVA (2-tailed probability value) was used with the Dunnett post hoc test for multiple groups using GraphPad Instat 3 software (San Diego, CA). The level of statistical significance was 0.05.

## Results

### Diabetes induction by streptozotocin

**Figure 1:**
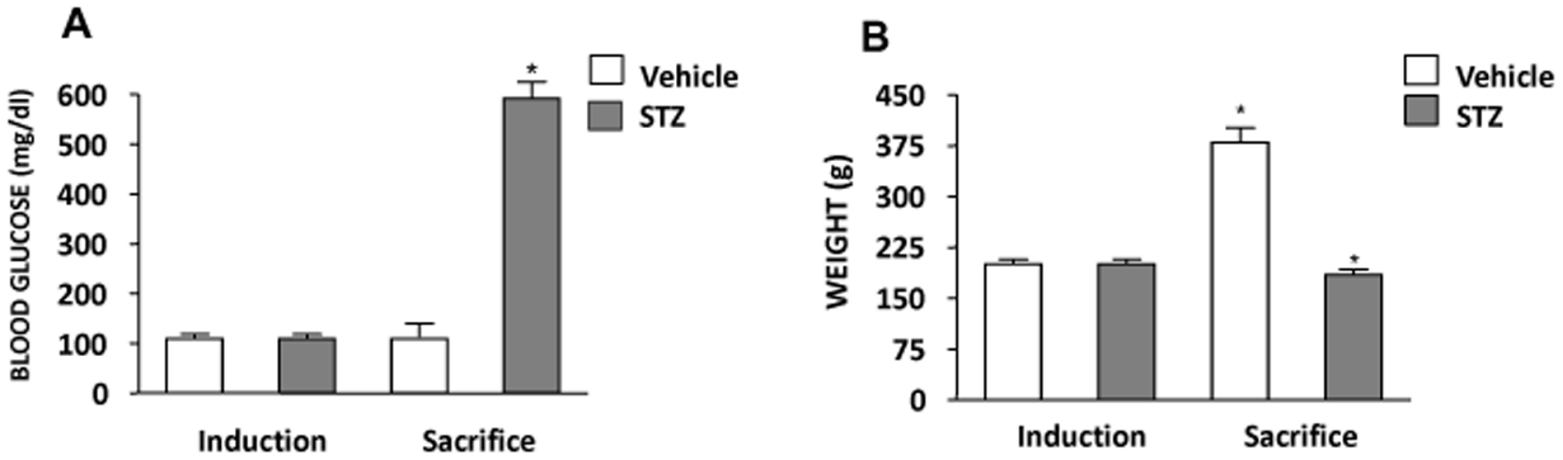
Experimental diabetes model. (**A**) Glycemia values and (**B**) body weight at baseline and 4 weeks post diabetes induction using 65 mg/Kg streptozotocin (STZ) in 2-months-old Sprague-Dawley rats. Values are mean ± SEM (n = 6). *p<0.0001 vs control.

Four weeks after STZ injection, diabetic animals had blood glucose 593 ± 14 mg/dl (animals were considered diabetic when blood glucose exceeds 300 mg/dl) and weighed 185 ± 8 g; in contrast, control animals had significantly lower of blood glucose levels (113 ± 7 mg/dl) and higher body weight (350 ± 22 g) as compared with diabetic animals (Fig. 1).

### SIRT1 detection and localization in VSMCs

**Figure 2:**
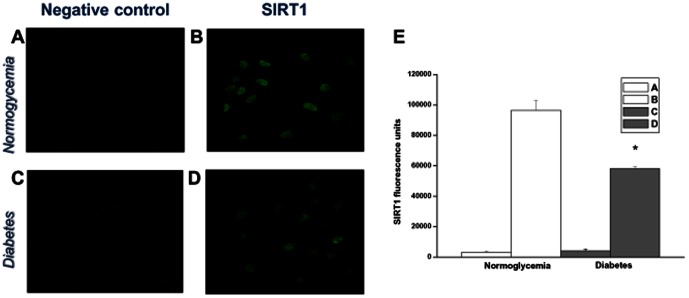
SIRT1 immuncytochemical detection in VSMCs from normoglycemic (A, B) and diabetic (C, D) rats. VSMCs were seeded (30,000/well) at passage 6 in 24-well plates. Two days after plating cells were probed with rabbit antibody to SIRT1 (1∶200) and then incubated with a secondary phycoerythrin-conjugated anti-rabbit antibody (1∶500) for 30 min at room temperature. VSMCs that received secondary antibody but no SIRT1 primary antibody were used as a negative control. 60× magnification. (**E**) Densitometric analyses of 3 separate experiments using ImageJ. * p<0.05 vs normoglycemia.

Once ascertained the purity of VSMC cultures using α-actin immunohistochemistry (data not shown), the presence and localization of SIRT1 was investigated. VSMCs from normoglycemic and diabetic rats were probed with a rabbit antibody for SIRT1 and then all incubated with a secondary anti-rabbit antibody conjugated to phycoerythrin. While SIRT1 was found to be prevalently localized in the nucleus, labeling appeared to be weaker in VSMCs from diabetic (Fig. 2D) as compared with control rats (Fig. 2B). This was confirmed by densitometric analysis of replicate images (Fig. 2E).

### Diabetes and high glucose downregulate SIRT1 protein

**Figure 3:**
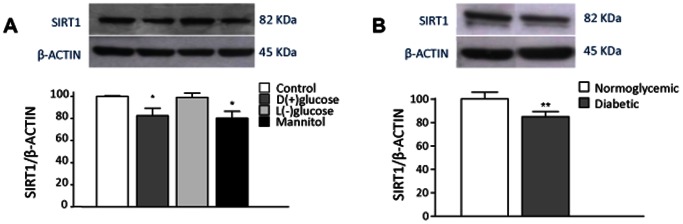
Effect of diabetes and high glucose on SIRT1 protein. (**A**) VSMCs from normoglycemic and STZ-diabetic rats were incubated in 10% FBS culture medium for 24 h. (**B**) VSMCs from normoglycemic rats were incubated in 10% FBS culture medium (control) for 24 h in the presence or absence of D-(+)-glucose, L-(−)-glucose and the osmotic regulator mannitol (all 22.2 mM). SIRT1 is shown as% expression, where 100% refers to the amount detected in untreated VSMCs from normoglycemic rats. Representative Western blot experiments are shown above bars. Values are mean ± SEM (n = 6–12). *p<0.05 vs control.

The aim of these experiments was to assess the effects of exposure to high glucose concentrations on the amount of SIRT1 protein both in the *ex vivo* STZ model and *in vitro* to dissect potential differential contributions of the diabetic condition *in toto* as opposed to the leading hyperglycemic status. As shown in Fig. 3A, levels of SIRT1 in diabetic VSMCs were significantly lower than in normoglycemic VSMCs. A similar experiment was performed in normoglycemic VSMCs in the presence of high concentrations (22.2 mM) of D-(+)-glucose, L-(−)-glucose and mannitol. As shown in Fig. 3B, incubation with D-(+)-glucose downregulated SIRT1 levels to a similar extent as that seen in diabetic VSMCs. Whereas the inactive isomer L-(−)-glucose did not affect SIRT1 levels, mannitol unexpectedly reduced SIRT1 levels and therefore may not be regarded simply as an osmotic control in this experiment design.

### SIRT1 modulation by TNF-α

**Figure 4:**
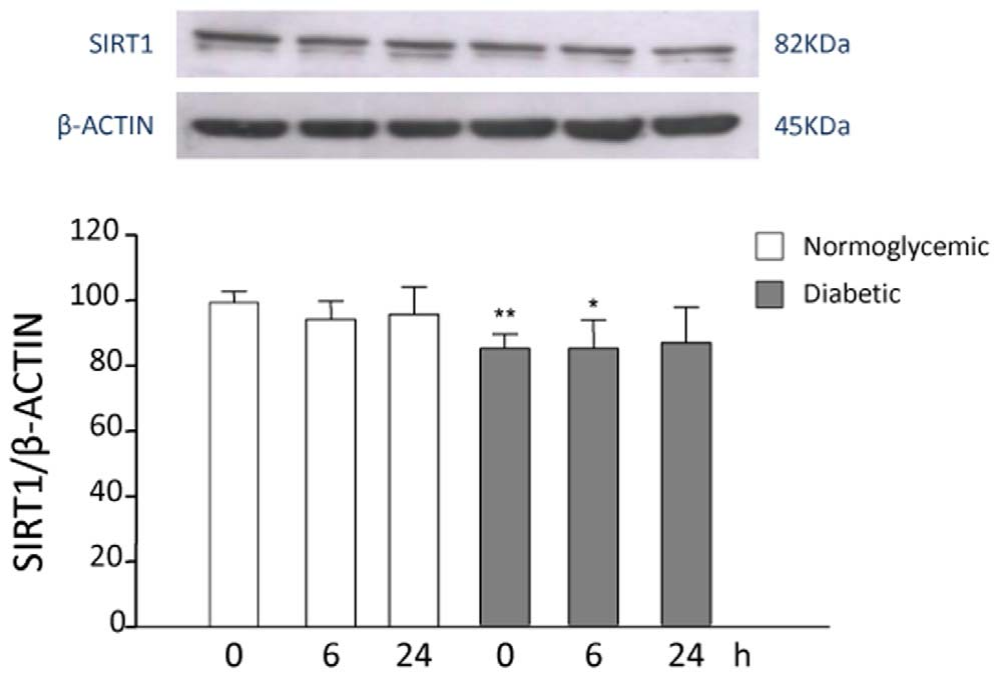
SIRT1 modulation under inflammatory stimulus by TNF-α. VSMCs from normoglycemic and STZ-diabetic rats were incubated with 30 ng/ml TNF-α for the indicated times; the amount of SIRT1 at baseline in normoglycemic VSMCs was taken as 100%. A representative Western blot is shown. Values are mean ± SEM (n = 6). *p<0.05, **p<0.01 vs control.

In previous studies, the pro-inflammatory cytokine TNF-α was shown to upregulate SIRT1 expression in a time-dependent manner in VSMCs [17]. We repeated this experiment in both VSMC groups. In our hands, no significant changes in SIRT1 protein levels were observed in response to TNF-α stimulation in normoglycemic and diabetic VSMCs at different time points (Fig. 4).

### Effects of 17β-estradiol on SIRT1 levels in different cell types

**Figure 5:**
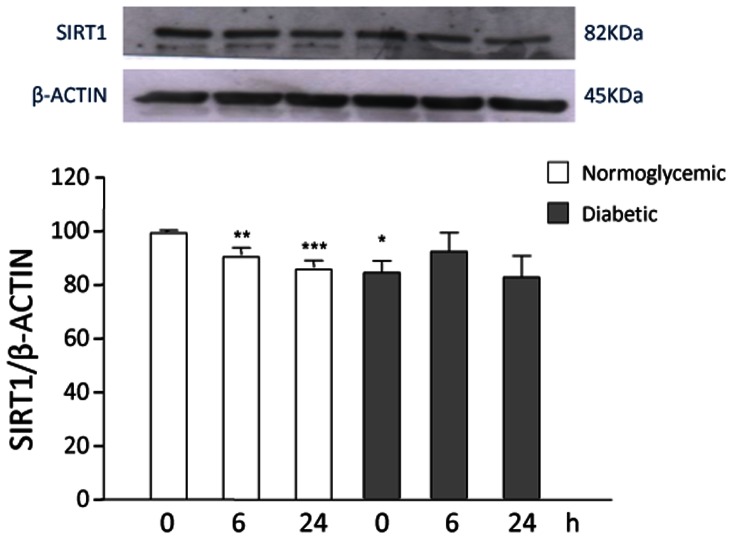
Effect of 17β-estradiol on SIRT1 levels in VSMCs. VSMCs from normoglycemic and STZ-diabetic rats were incubated with 10 nM 17β-estradiol for 0, 6 and 24 h in 2% FBS phenol-red free culture medium, to avoid any interference from the weak estrogenic agent phenol red. SIRT amounts in normoglycemic VSMCs at baseline were taken as 100%. A representative Western blot is shown. Values are mean ± SEM (n = 6). *p<0.05, **p<0.01, ***p<0.001 vs control.

**Figure 6:**
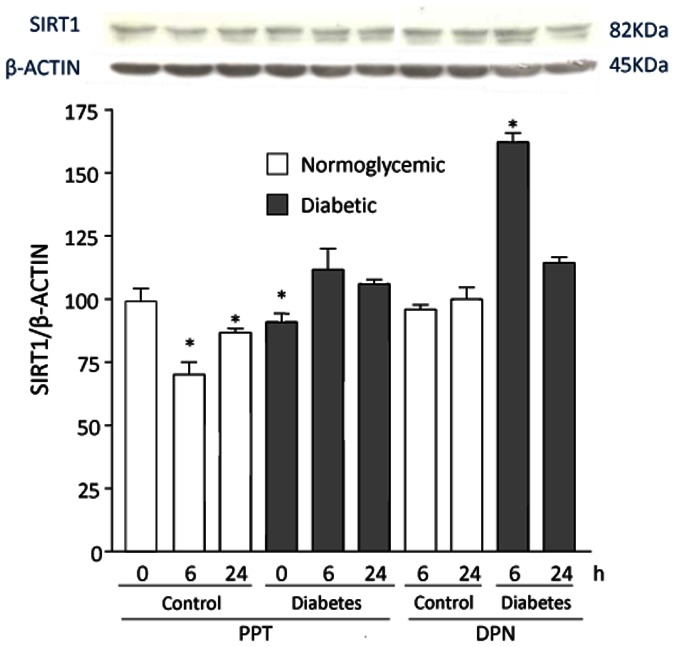
Effect of the selective ERα agonist PPT and the selective ERβ agonist DPN on SIRT1 levels in VSMCs. VSMCs from normoglycemic and STZ-diabetic rats were incubated with PPT or DPN (0,1 µM) for 0, 6 and 24 h in 2% FBS phenol-red free culture medium, to avoid any interference from the weak estrogenic agent phenol red. SIRT amounts in normoglycemic VSMCs at baseline were taken as 100%. A representative Western blot is shown. Values are mean ± SEM (n = 3). *p<0.05 vs control.

Based on previous studies from our group [14], [15] showing that the anti-inflammatory activity of 17β-estradiol is impaired in VSMCs from diabetic rats, we tested the hypothesis that SIRT1 could be a downstream target of the hormone in vascular cells. In normoglycemic VSMCs, incubation with 10 nM 17β-estradiol led to diminished SIRT1 accumulation during the time course (Fig. 5). Consistent with the findings shown in Figs 2 to 4 above, SIRT1 protein amount was lower in diabetic than in normoglycemic VSMCs at baseline. Conversely, 17β-estradiol did not induce further changes in SIRT1 protein amounts in diabetic VSMCs as compared with baseline (Fig. 5). 17β-estradiol is a nonselective estrogen receptor (ER) agonist. Therefore, to explore the estrogen signaling pathways involved in SIRT1 modulation, we performed experiments using the selective ERα agonist PPT and the selective ERβ agonist DPN. As shown in Fig. 6, treatment with PPT for 6 h and 24 h reduced SIRT1 levels in normoglycemic but not in diabetic VSMCs. By contrast, treatment with DPN had no effect on SIRT1 in normoglycemic VSMCs, whereas it increased SIRT1 levels in diabetic VSMCs after 6 h treatment, suggesting that 17β-estradiol modulated SIRT1 abundance mainly through ERα signaling.

**Figure 7:**
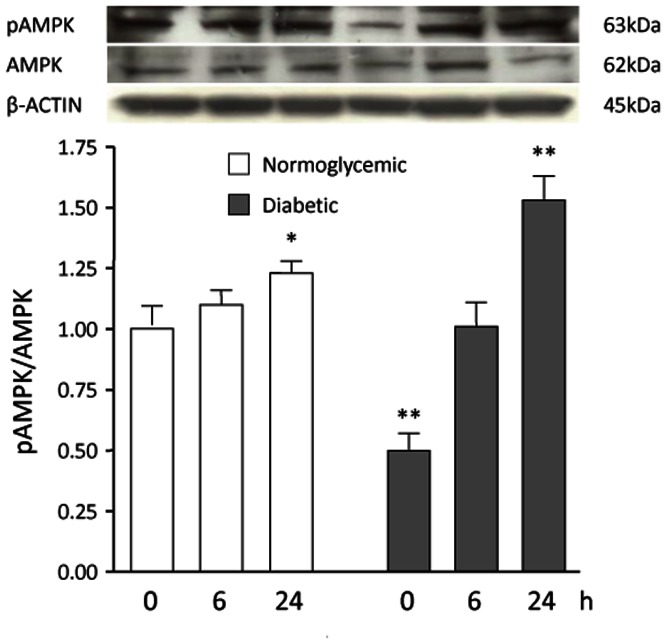
Effect of 17β-estradiol on total and phosphorylated AMP-activated protein kinase (AMPK) abundance in VSMCs. VSMCs from normoglycemic and STZ-diabetic rats were incubated with 10 nM 17β-estradiol for 0, 6 and 24 h in 2% FBS phenol-red free culture medium, to avoid any interference from the weak estrogenic agent phenol red. AMPK and pAMPK amounts in normoglycemic VSMCs at baseline were taken as 100%. A representative Western blot is shown. Values are mean ± SEM (n = 3). *p<0.05 vs control.

Because a close interplay between SIRT1 and AMP-activated protein kinase (AMPK) has been described in the regulation of cellular metabolism and inflammation [18], and because 17β-estradiol regulates the functional expression of AMPK [19], we measured the total expression and phosphorylation level of AMPK. The pAMPK to AMPK ratio was significantly reduced in VSMCs from diabetic as compared with normoglycemic animals (Fig. 7), in agreement with previous studies [20]. After treatment with 17β-estradiol, the total expression of AMPK remained unaltered, whereas levels of pAMPK were significantly increased by 17β-estradiol in both VSMC groups (Fig. 7). Thus, 17β-estradiol–mediated SIRT1 downregulation was associated with increased AMPK activation.

**Figure 8:**
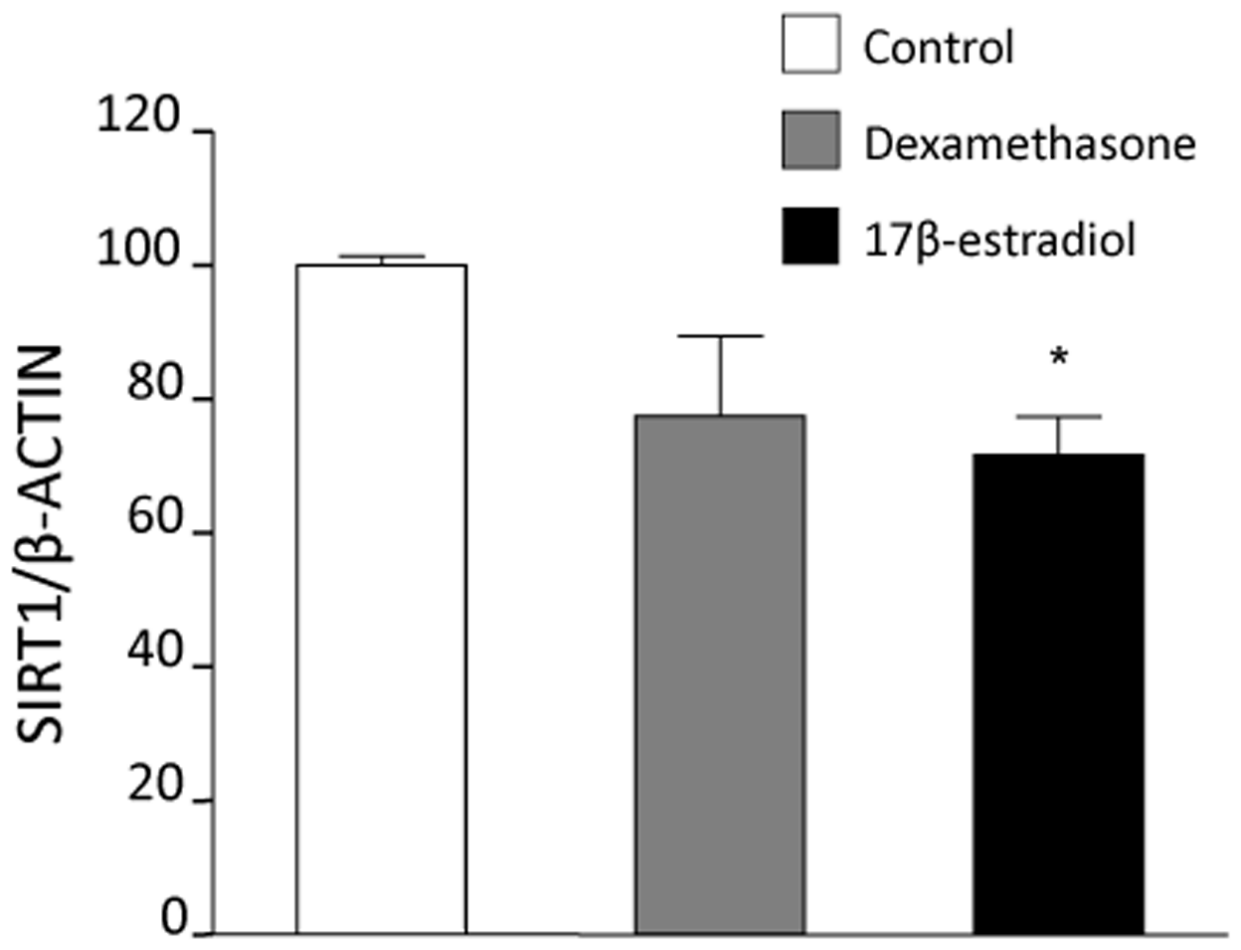
SIRT1 regulation by steroid hormone agents in freshly isolated human peripheral blood mononuclear cells (PBMCs). Cells were incubated with 10 nM dexamethasone or 10 nM 17ß-estradiol for 24 h. SIRT1 amounts in untreated PBMCs were taken as 100%. Values are mean ± SEM (n = 4). *p<0.01 vs control.

Finally, we tested whether the above pattern of SIRT1 regulation by 17β-estradiol could apply to human cells. As a test model we chose PBMCs in view of the association between Sirt1 gene expression in this cell type and metabolic control [12]. Incubation with 17β-estradiol for 24 h significantly reduced SIRT1 protein amounts in human PBMCs (Fig. 8). While the trend was similar, changes in SIRT1 in response to treatment with 10 nM dexamethasone, a well-established anti-inflammatory agent, did not achieve statistical significance.

## Discussion

SIRT1 plays an important role in many pathophysiological processes, including cellular senescence/aging and inflammation [3], [21], yet the precise role of SIRT1 in oxidative stress remains enigmatic. Studies in multiple organs and cell systems hint that SIRT1 depletion could be an adaptive mechanism to promote oxidative stress resistance. However, loss of SIRT1 was also interpreted to contribute to the detrimental effects of oxidative stress [22], [23]. Some of the seemingly conflicting results that have been reported may be attributable to an optimal window for SIRT1 activity, as has been observed for oxidative stress levels in the heart [24]. At the same time, amelioration of oxidative stress in other cell types and organs suggests that SIRT1 activation could have a real therapeutic benefit under conditions of excessive ROS production. There is evidence that SIRT1 can suppress ROS generation by virtue of its anti-inflammatory actions. NAD(P)H oxidase is an important producer of ROS *in vivo*, and SIRT1 can inhibit production of an upstream signal, TNF-α, through deacetylation and inhibition of nuclear factor (NF)-κB in macrophages [25]. By contrast, the pharmacologic inhibition of sirtuins has also been reported to inhibit the activation of the NF-κB pathway and decrease the production of LPS-induced cytokines in J774 macrophages *in vitro* [10]. Our TNF-α stimulation experiments in VSMCs yielded no hint of SIRT1 modulation, in contrast to findings by Zhang and coworkers [17]. The discrepancy may be due to variability in cell culture conditions and/or TNF-α bioactivity compared with those of Zhang et al. It is however possible that other inflammatory stimuli combined with TNF-α induce upregulation of SIRT1, which may be part of a protective response of VSMCs to inflammatory events.

In agreement with previous studies in human endothelial cells and STZ-diabetic mice [4], SIRT1 accumulation fell in diabetic compared to normoglycemic VSMCs, as confirmed using two different experimental procedures, and our *in vitro* high glucose studies are consistent with this pattern. To the best of our knowledge, this is the first demonstration of reduced SIRT1 levels in aortic VSMCs from STZ-diabetic rats. SIRT1 is also an important regulator of macrophage inflammatory responses in the context of insulin resistance [26]. Because SIRT1 requires NAD for its enzymatic activity [27], a decline in NAD biosynthesis on high glucose or hyperglycemia may result in a significant reduction of SIRT1 levels and subsequent loss of the glucose- or insulin-responsive phenotypes. Accordingly, a significant reduction in NAD+ levels and SIRT1 activity in physiologically aged female Wistar rats has been recently reported [28]. Similarly, although estrogen has been shown to inhibit VSMC proliferation under normal glucose concentrations, high glucose conditions abolish the antiproliferative effect of estrogen through as yet unknown mechanisms [29].

The relationship between SIRT1 and estrogen signaling is still controversial. In particular, it is unclear whether SIRT1 actually serves as an estrogen receptor (ER)α co-activator or co-repressor in oncogenesis [11], [30]. To the best of our knowledge, we here report for the first time that 17β-estradiol reduced the expression of SIRT1 in normoglycemic VSMCs, and did so in human PBMCs as well, whereas it did not appear to affect SIRT1 protein amounts in diabetic VSMCs. SIRT1 downregulation was mimicked by the selective ERα agonist PPT, but not the selective ERβ agonist DPN, which in turn increased SIRT1 abundance after short-term incubation in VSMCs from diabetic animals. Previous studies from our laboratory showed that VSMCs from STZ-diabetic rats overexpress ERβ compared with normoglycemic animals [15]. This is associated with impaired downregulation of the inflammatory enzyme inducible NO synthase by 17β-estradiol [15]. Thus, ERα and ERβ appear to differentially regulate SIRT1, consistent with a *yin-yang* action pattern in tissues where both isoforms are co-expressed and with a recent study showing that SIRT3 protein levels are affected by the ERα/ERβ ratio in breast cancer specimens [31]. In addition, SIRT1 has been described to reverse the acetylation of ERα [32] and thereby reduce its transcriptional activity, although the relevance of this mechanism was not tested in the present study. A potential involvement of GPER, a non-genomic mediator of estrogen action expressed in VSMC [33], in SIRT1 modulation by 17β-estradiol remains to be determined.

An additional finding from the present study is that 17β-estradiol treatment enhanced AMPK activation in VSMCs from normoglycemic and reversed the significant impairment of AMPK activity in those from diabetic animals compared with controls, in line with previous studies [20]. It cannot be ruled out that the discordant modulation of SIRT1 and AMPK proteins by 17β-estradiol in VSMCs occurred through independent mechanisms. However, 17β-estradiol is a known activator of AMPK [19], and non-concomitant regulation of AMPK activity and SIRT1 abundance has been reported in different tissues [34]–[36]. Thus, 17β-estradiol-mediated SIRT1 downregulation may be a potential downstream effect of AMPK activation resulting from amelioration of oxidant burden or changes in energy state [18], [36].

In conclusion, the present study shows that previous diabetes induction *in vivo* negatively regulated SIRT1 amounts in rat VSMCs, consistent with the *in vitro* effects of high glucose concentrations, whereas we were unable to demonstrate SIRT1 modulation by TNF-α. In addition, 17β-estradiol decreased SIRT1 levels in VSMCs from normoglycemic but not diabetic rats most likely through ERα signaling, possibly as a downstream effect of AMPK activation. This negative regulation of SIRT1 could also be observed in human cell types, namely PBMCs freshly isolated from buffy coat, suggesting that findings in the rodent cell model can be translated to human settings. Whether the observed changes in SIRT1 protein levels in VSMCs are paralleled by changes in protein deacetylase activity remains to be determined. Future studies should also explore how the different levels of the SIRT1 regulatory network are connected in response to diverse cellular stressors, and what balances the net SIRT1 activity [9].

## References

[1] Ungvari Z, Kaley G, de Cabo R, Sonntag WE, Csiszar A (2010) Mechanisms of vascular aging: new perspectives. J Gerontol A Biol Sci Med Sci 65: 1028–1041. 20576649 10.1093/gerona/glq113 PMC2950814

[2] Li L, Zhang HN, Chen HZ, Gao P, Zhu LH, et al (2011) SIRT1 acts as a modulator of neointima formation following vascular injury in mice. Circ Res 108: 1180–1189. 21474819 10.1161/CIRCRESAHA.110.237875

[3] Guarente L (2011) Franklin H. Epstein Lecture: Sirtuins, aging, and medicine. N Engl J Med 364: 2235–2244. 21651395 10.1056/NEJMra1100831

[4] Orimo M, Minamino T, Miyauchi H, Tateno K, Okada S, et al (2009) Protective role of SIRT1 in diabetic vascular dysfunction. Arterioscler Thromb Vasc Biol 29: 889–894. 19286634 10.1161/ATVBAHA.109.185694

[5] Banks AS, Kon N, Knight C, Matsumoto M, Gutiérrez-Juárez R, et al (2008) SirT1 gain of function increases energy efficiency and prevents diabetes in mice. Cell Metab 8: 333–341. 18840364 10.1016/j.cmet.2008.08.014 PMC3222897

[6] Miyazaki R, Ichiki T, Hashimoto T, Inanaga K, Imayama I, et al (2008) SIRT1, a longevity gene, downregulates angiotensin II type 1 receptor expression in vascular smooth muscle cells. Arterioscler Thromb Vasc Biol 28: 1263–1269. 18420994 10.1161/ATVBAHA.108.166991

[7] Tajbakhsh N, Sokoya EM (2012) Regulation of cerebral vascular function by sirtuin 1. Microcirculation 19: 336–342. 22313457 10.1111/j.1549-8719.2012.00167.x

[8] Feige JN, Auwerx J (2008) Transcriptional targets of sirtuins in the coordination of mammalian physiology. Curr Opin Cell Biol 20: 303–309. 18468877 10.1016/j.ceb.2008.03.012 PMC2447870

[9] Kwon HS, Ott M (2008) The ups and downs of SIRT1. Trends Biochem Sci 33: 517–525. 18805010 10.1016/j.tibs.2008.08.001

[10] Fernandes CA, Fievez L, Neyrinck AM, Delzenne NM, Bureau F, et al (2012) Sirtuin inhibition attenuates the production of inflammatory cytokines in lipopolysaccharide-stimulated macrophages. Biochem Biophys Res Commun 420: 857–861. 22469470 10.1016/j.bbrc.2012.03.088

[11] Moore RL, Dai Y, Faller DV (2012) Sirtuin 1 (SIRT1) and steroid hormone receptor activity in cancer. J Endocrinol 213: 37–48. 22159506 10.1530/JOE-11-0217 PMC3804056

[12] de Kreutzenberg SV, Ceolotto G, Papparella I, Bortoluzzi A, Semplicini A, et al (2010) Downregulation of the longevity-associated protein sirtuin 1 in insulin resistance and metabolic syndrome: potential biochemical mechanisms. Diabetes 59: 1006–1015. 20068143 10.2337/db09-1187 PMC2844808

[13] Wei M, Ong L, Smith MT, Ross FB, Schmid K, et al (2003) The streptozotocin-diabetic rat as a model of the chronic complications of human diabetes. Heart Lung Circ 12: 44–50. 16352106 10.1046/j.1444-2892.2003.00160.x

[14] Zancan V, Santagati S, Bolego C, Vegeto E, Maggi A, et al (1999) 17β-estradiol decreases nitric oxide synthase II synthesis in vascular smooth muscle cells. Endocrinology 140: 2004–2009. 10218948 10.1210/endo.140.5.6694

[15] Maggi A, Cignarella A, Brusadelli A, Bolego C, Pinna C, et al (2003) Diabetes undermines estrogen control of inducible nitric oxide synthase function in rat aortic smooth muscle cells through overexpression of estrogen receptor-β. Circulation 108: 211–217. 12821541 10.1161/01.CIR.0000079311.39939.94

[16] Idel S, Ellinghaus P, Wolfrum C, Nofer JR, Gloerich J, et al (2002) Branched chain fatty acids induce nitric oxide-dependent apoptosis in vascular smooth muscle cells. J Biol Chem 277: 49319–49325. 12368296 10.1074/jbc.M204639200

[17] Zhang HN, Li L, Gao P, Chen HZ, Zhang R, et al (2010) Involvement of the p65/RelA subunit of NF-κB in TNF-α-induced SIRT1 expression in vascular smooth muscle cells. Biochem Biophys Res Commun 397: 569–575. 20617556 10.1016/j.bbrc.2010.05.160

[18] Ruderman NB, Xu XJ, Nelson L, Cacicedo JM, Saha AK, et al (2010) AMPK and SIRT1: a long-standing partnership? Am J Physiol Endocrinol Metab 298: E751–E760. 20103737 10.1152/ajpendo.00745.2009 PMC2853213

[19] Schulz E, Anter E, Zou MH, Keaney JF Jr (2005) Estradiol-mediated endothelial nitric oxide synthase association with heat shock protein 90 requires adenosine monophosphate-dependent protein kinase. Circulation 111: 3473–3480. 15967841 10.1161/CIRCULATIONAHA.105.546812

[20] Ning J, Xi G, Clemmons DR (2011) Suppression of AMPK activation via S485 phosphorylation by IGF-I during hyperglycemia is mediated by AKT activation in vascular smooth muscle cells. Endocrinology 152: 3143–3154. 21673100 10.1210/en.2011-0155 PMC3138225

[21] Yao H, Rahman I (2012) Perspectives on translational and therapeutic aspects of SIRT1 in inflammaging and senescence. Biochem Pharmacol 84: 1332–1339. 22796566 10.1016/j.bcp.2012.06.031 PMC3482299

[22] Wu A, Ying Z, Gomez-Pinilla F (2006) Oxidative stress modulates Sir2alpha in rat hippocampus and cerebral cortex. Eur J Neurosci 23: 2573–2580. 16817860 10.1111/j.1460-9568.2006.04807.x

[23] Caito S, Rajendrasozhan S, Cook S, Chung S, Yao H, et al (2010) SIRT1 is a redox-sensitive deacetylase that is post-translationally modified by oxidants and carbonyl stress. FASEB J 24: 3145–3159. 20385619 10.1096/fj.09-151308 PMC2923349

[24] Alcendor RR, Gao S, Zhai P, Zablocki D, Holle E, et al (2007) Sirt1 regulates aging and resistance to oxidative stress in the heart. Circ Res 100: 1512–1521. 17446436 10.1161/01.RES.0000267723.65696.4a

[25] Shen Z, Ajmo JM, Rogers CQ, Liang X, Le L, et al (2009) Role of SIRT1 in regulation of LPS- or two ethanol metabolites-induced TNF-α production in cultured macrophage cell lines. Am J Physiol Gastrointest Liver Physiol 296: G1047–G1053. 19299582 10.1152/ajpgi.00016.2009 PMC2696216

[26] Yoshizaki T, Schenk S, Imamura T, Babendure JL, Sonoda N, et al (2010) SIRT1 inhibits inflammatory pathways in macrophages and modulates insulin sensitivity. Am J Physiol Endocrinol Metab 298: E419–E428. 19996381 10.1152/ajpendo.00417.2009 PMC2838524

[27] Imai S, Armstrong CM, Kaeberlein M, Guarente L (2000) Transcriptional silencing and longevity protein Sir2 is an NAD-dependent histone deacetylase. Nature 403: 795–800. 10693811 10.1038/35001622

[28] Braidy N, Guillemin G, Mansour H, Chan-Ling T, Poljak A, et al (2011) Age related changes in NAD+ metabolism, oxidative stress and Sirt1 Activity in Wistar rats. PLOS ONE 6: e19194. 21541336 10.1371/journal.pone.0019194 PMC3082551

[29] Ortmann J, Veit M, Zingg S, Di Santo S, Traupe T, et al (2011) Estrogen receptor-α but not -β or GPER inhibits high glucose-induced human VSMC proliferation: potential role of ROS and ERK. J Clin Endocrinol Metab 96: 220–228. 20962025 10.1210/jc.2010-0943 PMC3038487

[30] Elangovan S, Ramachandran S, Venkatesan N, Ananth S, Gnana-Prakasam JP, et al (2011) SIRT1 is essential for oncogenic signaling by estrogen/estrogen receptor α in breast cancer. Cancer Res 71: 6654–6664. 21920899 10.1158/0008-5472.CAN-11-1446 PMC3206200

[31] Sastre-Serra J, Nadal-Serrano M, Gabriel Pons D, Valle A, Garau I, García-Bonafé M, Oliver J, Roca P. (2013) The oxidative stress in breast tumors of postmenopausal women is ERα/ERβ ratio dependent. Free Radic Biol Med doi:pii: S0891-5849(13)00099-3. 10.1016/j. 10.1016/j.freeradbiomed.2013.03.005 23499841

[32] Kim MY, Woo EM, Chong YT, Homenko DR, Kraus WL (2006) Acetylation of estrogen receptor alpha by p300 at lysines 266 and 268 enhances the deoxyribonucleic acid binding and transactivation activities of the receptor. Mol Endocrinol 20: 1479–1493. 16497729 10.1210/me.2005-0531 PMC1483068

[33] Prossnitz ER, Arterburn JB, Smith HO, Oprea TI, Sklar LA, et al (2008) Estrogen signaling through the transmembrane G protein-coupled receptor GPR30. Annu Rev Physiol 70: 165–190. 18271749 10.1146/annurev.physiol.70.113006.100518

[34] Gurd BJ, Yoshida Y, Lally J, Holloway GP, Bonen A (2009) The deacetylase enzyme SIRT1 is not associated with oxidative capacity in rat heart and skeletal muscle and its overexpression reduces mitochondrial biogenesis. J Physiol 587: 1817–1828. 19237425 10.1113/jphysiol.2008.168096 PMC2683967

[35] Boily G, Seifert EL, Bevilacqua L, He XH, Sabourin G, et al (2008) SirT1 regulates energy metabolism and response to caloric restriction in mice. PLoS One 3: e1759. 18335035 10.1371/journal.pone.0001759 PMC2258149

[36] Lempiäinen J, Finckenberg P, Levijoki J, Mervaala E (2012) AMPK activator AICAR ameliorates ischaemia reperfusion injury in the rat kidney. Br J Pharmacol 166: 1905–1915. 22324445 10.1111/j.1476-5381.2012.01895.x PMC3402813

